# Development of peer assessment rubrics in simulation-based learning for advanced cardiac life support skills among medical students

**DOI:** 10.1186/s41077-024-00301-7

**Published:** 2024-06-24

**Authors:** Sethapong Lertsakulbunlue, Anupong Kantiwong

**Affiliations:** Department of Pharmacology, Phramongkutklao College of Medicine, Bangkok, 10400 Thailand

**Keywords:** Simulation, Peer assessment, Medical student, Rubrics, ACLS, Validity, Reliability

## Abstract

**Introduction:**

Peer assessment can enhance understanding of the simulation-based learning (SBL) process and promote feedback, though research on its rubrics remains limited. This study assesses the validity and reliability of a peer assessment rubric and determines the appropriate number of items and raters needed for a reliable assessment in the advanced cardiac life support (ACLS) context.

**Methods:**

Ninety-five third-year medical students participated in the ACLS course and were assessed by two teachers (190 ratings) and three peers (285 ratings). Students rotated roles and were assessed once as a team leader on a ten-item rubric in three domains: electrocardiogram and ACLS skills, management and mechanisms, and affective domains. Messick’s validity framework guided the collection of validity evidence.

**Results:**

Five sources of validity evidence were collected: (1) content: expert reviews and alpha, beta, and pilot tests for iterative content validation; (2) response process: achieved acceptable peer interrater reliability (intraclass correlation = 0.78, *p* = 0.001) and a Cronbach’s alpha of 0.83; (3) internal structure: demonstrated reliability through generalizability theory, where one peer rater with ten items achieved sufficient reliability (Phi-coefficient = 0.76), and two raters enhanced reliability (Phi-coefficient = 0.85); construct validity was supported by confirmatory factor analysis. (4) Relations to other variables: Peer and teacher ratings were similar. However, peers rated higher in scenario management; further generalizability theory analysis indicated comparable reliability with the same number of teachers. (5) Consequences: Over 80% of students positively perceived peer assessment on a 5-point Likert scale survey.

**Conclusion:**

This study confirms the validity and reliability of ACLS SBL rubrics while utilizing peers as raters. Rubrics can exhibit clear performance criteria, ensure uniform grading, provide targeted feedback, and promote peer assessment skills.

**Supplementary Information:**

The online version contains supplementary material available at 10.1186/s41077-024-00301-7.

## Introduction

### Simulation-based learning

Simulation, an innovative instrument within medical education, serves as a practical emulation of scenarios or events designed for the purposes of learning, assessment, or research [1, 2]. With the expansion of medical knowledge and limited training time, simulation is increasingly seen as a bridge between the traditional apprenticeship model and the need for skill training in modern medicine [2]. A realistic simulation-based learning (SBL) scenario allows medical students to develop skills using mannikin or tools before real patient interaction, enhancing their clinical and related skills [3]. SBL provides a safe, controlled environment that enriches experiences and boosts medical students’ confidence and decision-making abilities [4].

### Simulation-based learning content at Phramongkutklao College of Medicine

Recognizing the benefits of simulation-based learning in ACLS and electrocardiogram (EKG) education, Phramongkutklao College of Medicine (PCM) developed a specialized course. This course enrolls about 100 preclinical medical students and focuses on EKG interpretation and ACLS skills within realistic scenarios using high-fidelity mannequins to enhance student learning. The course also integrated peer assessment using scoring rubrics to enhance engagement further.

### Peer assessment benefits and limitations

Engaging students and keeping them focused on learning objectives and assessment criteria pose significant challenges in large classes. Furthermore, feedback implementation often suffers from inadequate teacher participation [5]. Peer assessment enhances learning objectives and feedback comprehension by allowing students to assess each other’s work against defined criteria, thus offering cognitive and pedagogical benefits while promoting autonomy and engagement [6–8]. This method increases group involvement and motivation, supported by self-determination theory, by fostering students’ autonomy, competence, and relatedness [6, 9]. Additionally, acting as peer assessors encourages students to utilize higher-order thinking skills for analyzing peers’ performances, leading to improved learning outcomes in alignment with Bloom’s taxonomy [10, 11].

Peer assessment serves both formative and summative roles, enhancing student confidence and may reduce the needed number of teachers involved in the formative stages [12]. Feedback, integral to learning, often lacks effective implementation, attributed to limited teacher participation [5]. Hence, peer-led formative exams not only assist learners’ feedback needs [13] but also, through continuous application, bolster knowledge retention and future performance [14]. However, peer assessment reliability and validity are concerns, especially in summative contexts, due to potential bias [15]. The primary focus is validity, specifically the agreement between peer and teacher assessments [15, 16]. Despite teacher ratings being considered more valid traditionally, some studies suggest that peer assessments could be more accurate [13].

### Rubrics development

Rubrics have gained popularity in peer assessment as they delineate explicit performance criteria and expectations, ensure uniformity in grading and assessment, provide targeted feedback on students’ strengths and weaknesses, and promote the development of peer assessment competencies [17]. Scoring rubrics fall into two categories, including analytic and holistic. Analytic rubrics break down the response into key components, assigning points to each. Holistic rubrics assess the overall quality of the response, categorizing student work based on its quality level [18]. In line with outcome-based education, rubrics are designed to display the competencies specified in the learning objectives [19]. Consequently, an analytical framework derived from the Association for Medical Education in Europe (AMEE) guidelines underpins the rubric development process. This framework ensures a detailed delineation of competencies, encompassing cognitive, psychomotor, and affective domains and sub-competencies—such as EKG interpretation and communication skills—aligned with the specified learning objectives [20]. Each rubric criteria can then be synthesized according to the expected expertise level.

### Messick’s validity framework

In validating the rubric, the study employed Messick’s validity framework to guide and ensure the validity of this research [21–23]. The framework outlines a systematic method to obtain construct validity evidence, with Messick highlighting five essential aspects: (1) content, ensuring alignment of test items with the intended construct; (2) response process, prioritizing data integrity and clear instructions; (3) internal structure, examining the exam’s psychometric properties; (4) relations with other variables, analyzing theoretical correlations; and (5) consequences, determining effects on learners, instructors, and the system [21].

Methods to ensure content validity involve using established instruments and conducting expert reviews of draft items. For the response process, clear instructions for candidates and comprehensive rater training are essential. Internal structure is assessed through measures like Cronbach’s alpha, inter-rater reliability, and generalizability theory, focusing on the psychometric properties. The relationships with other variables and the consequences are analyzed through correlations between survey scores and external variables and by exploring short-term pass rates, standard settings, and stakeholder perceptions, respectively [21, 22, 24].

### Gap of knowledge and objectives

Despite the recognized advantages of SBL, there is a scarcity of data on the validity and reliability of assessment rubrics for ACLS programs that incorporate peer assessment. Existing research on the reliability of applying generalizability theory to determine the variances of assessment scores in SBL rarely incorporates clinical scenarios and tends to emphasize the number of assessments and raters, often constrained by small class sizes [25, 26]. In contrast, this study involves a one-time assessment for each student, dictated by resource limitations among 100 participants. Moreover, prior investigations into peer versus teacher assessment in SBL have been limited to classical test theory [27, 28]. This indicates a research gap in determining the ideal number of peer raters to achieve reliable assessment for the SBL context.

While SBL discloses many advantages, it is important to note that it requires a significant number of teachers and extensive resources. Additionally, the performance-based nature of SBL presents a challenge in accurately assessing student performance and developing rubrics that balance simplicity for student use and the requisite complexity for comprehensive assessment [29]. Hence, this study intends to estimate the validity and reliability of rubrics when raters are peers, giving important insights for creating assessments of medical students’ ACLS SBL. This study also seeks to identify the optimal number of items and raters required for reliable assessments. The availability of physicians or academics as raters is especially important for medical schools with low resources. In addition to contributing feedback in the cognitive, psychomotor, and affective domains, peers can support teachers by conducting useful assessments in formative settings or leading peer-led mock exams.

## Methods

### Content

This study’s report on the methodology of simulation-based learning interventions was aligned with the CONSORT statement and its extension for simulation-based research reporting (Supplementary Table 1) [30, 31].

#### Simulation-based learning course

**Fig. 1:**
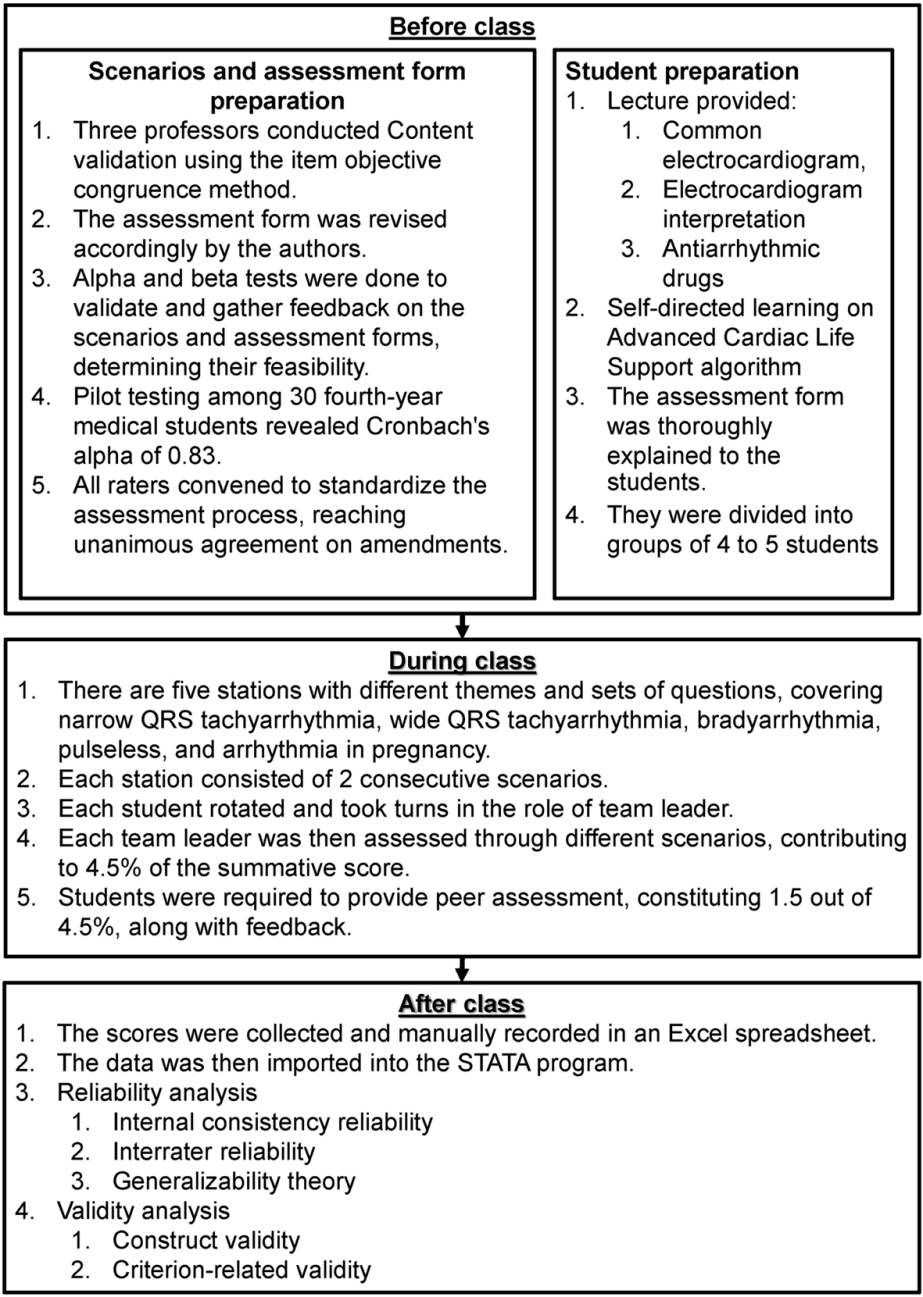
Study flow of the integration of simulation-based learning into clinically correlated electrocardiogram interpretation and ACLS course

A simulation-based course integrating EKG interpretation within an ACLS station was conducted for 95 third-year medical students, focusing on three main objectives: (1) interpreting standard EKG in ACLS scenarios; (2) providing verbal care aligned with the ACLS algorithm; and (3) understanding the pharmacological treatments’ mechanisms in ACLS, as shown in Fig. 1. Students engaged in diverse simulated case studies mirroring real-life scenarios and adhering to the difficulty of the Thai Medical Competency Assessment Criteria [32]. The simulations employed high-fidelity mannequins to facilitate basic physical examinations and provide a realistic experience, covering themes such as tachyarrhythmia, bradyarrhythmia, asystole, myocardial infarction, and pregnancy-related arrhythmia. Moreover, peer assessment with a standardized rubric was used to enhance engagement and comprehension of the objectives.

#### Assessment rubrics development and content review

AMEE guiding framework and literature on EKG interpretation assessment formats were reviewed to aid the scoring rubric development [20, 33]. The rubrics had three domains aligning with the class objectives: (1) EKG and ACLS algorithm skills, (2) management and mechanisms of action, and (3) affective domains. It consisted of 10 items, with a maximum score of 10 points for each. The developed holistic rubric allowed raters to judge the work based on the criteria outlined for the best-fit level. In addition to keeping the assessment form simple, this ameliorates peers’ capacity to give comments at different levels and estimate performance levels arbitrarily.

Rubric-guided questions are gauged using a rating scale of 10, 8, 6, 4, 2, and 0, ensuring the continuous rating scale. The assessment form included the following items: (1) sequential interpretation of EKG, (2) accurate interpretation of EKG, (3) diagnosis of EKG, (4) sequence of ACLS algorithm, (5) scenario management, (6) accurate pharmaceutical treatment, (7) pharmacological mechanism of action, (8) interpersonal skills, (9) communication skills, and (10) learning responsibility. Two teachers and three peer raters rated each team leader. Assessments are carried out in paper-based formats to improve validity and reliability, with assessors and assessees being randomly assigned. The assessments are non-anonymous to ensure transparency. Furthermore, the study includes qualitative comments to deepen the assessment process [13].

**Fig. 2:**
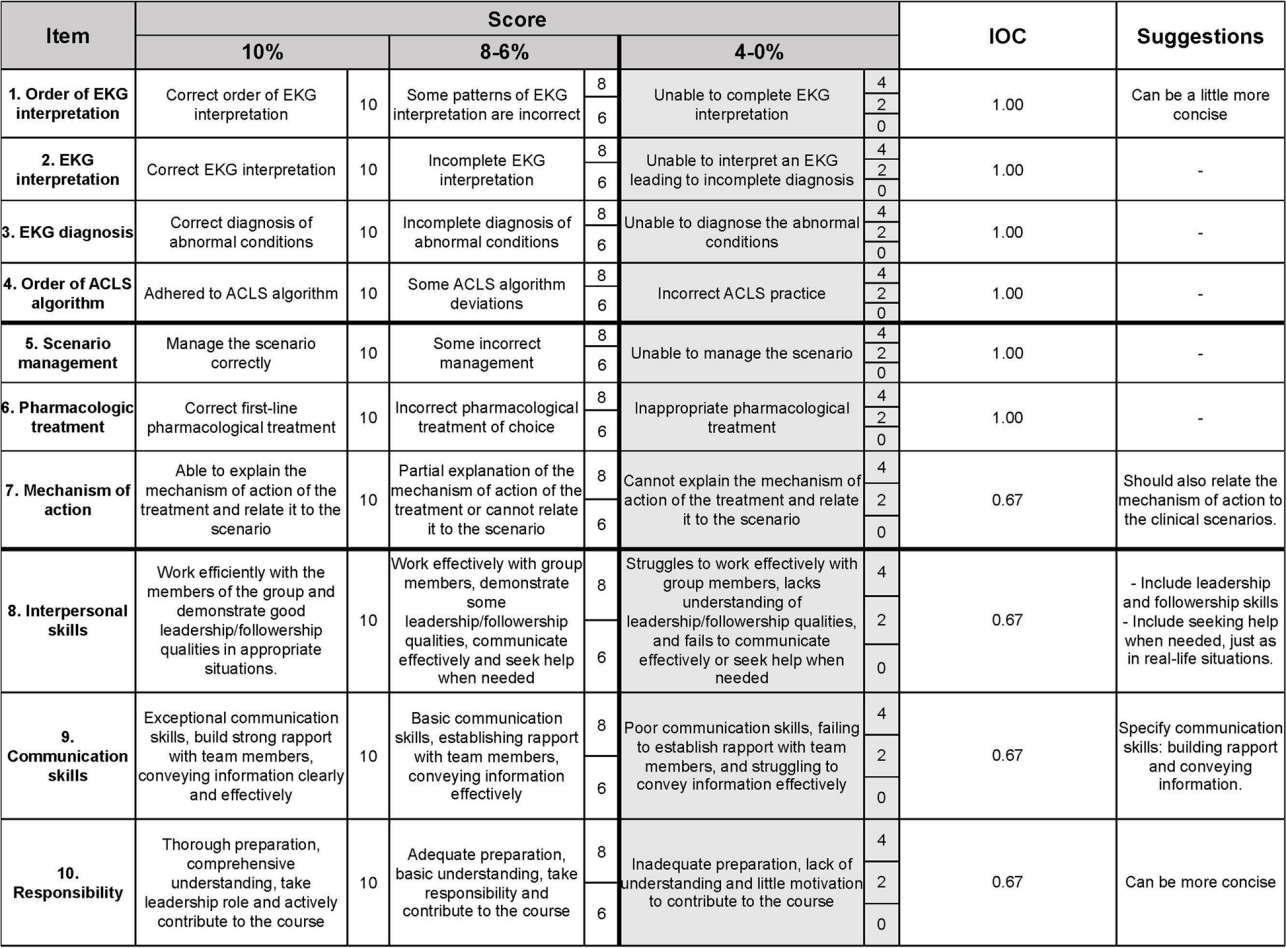
English version of the assessment rubric used in the integration of simulation-based learning into clinically correlated electrocardiogram interpretation and ACLS course. IOC, item objective congruence index

Before the class, three expert instructors reviewed the content of the assessment form to ensure alignment with the learning objectives and feasibility for use, employing the item objective congruence (IOC) approach [34]. The authors appropriately updated the form prior to its implementation. Figure 2 shows the entire assessment form with suggestions from the reviewer.

#### Alpha, *beta*, and pilot tests

To ensure the scenario’s feasibility, difficulty and assessment rubric content, alpha, beta, and pilot tests were conducted. Two intern doctors participated in the alpha test, currently a Phramongkutklao College of Medicine teacher assistant and previously earned an ACLS certificate. Then, five fourth-year medical students took the beta test. The authors then fine-tuned the scenarios as suggested by the emergency medicine staff. Next, a pilot exam was conducted among 30 fourth-year medical students who had taken an ACLS course the year before. With two teacher raters, the response showed a Cronbach’s alpha of 0.83. Hence, this proves the internal consistency and reliability of the assessment form. These students had previously studied comparable lectures on antiarrhythmic medications. However, they conducted self-directed learning (SDL) on the ACLS algorithm and standard EKG cases. They were assessed using five distinct case scenarios under examination conditions, which were thematically similar to those carried out for the current study’s participants.

### Response process

#### Student preparation and peer assessment preparation

The students were assigned to groups of 10, divided into teams of 4 to 5 members based on their student identification numbers. All students were briefed and assigned SDL tasks. These tasks involved exercises on interpreting common EKG outlined in the ACLS algorithm and studying the ACLS algorithm guidelines and steps for EKG interpretation. After this, lectures on relevant medications and normal and abnormal EKG interpretation were delivered. The assessment rubrics were comprehensively introduced to every student before the course. This was followed by a video of the beta tests done in the previous year, and the teacher demonstrated the use of the rubric.

#### Teacher assessment preparation

All raters participated in a conference to standardize the assessment procedure and gain a thorough understanding of the class objectives and assessment form. All raters approved the assessment forms in unanimity. Furthermore, all raters had experience applying the rubrics in the pilot testing phase.

#### Assessment process

Five stations, each giving two successive scenarios, were set up for the class. In every scenario, a team of four to five students was tested, and only the team leader’s performance was judged in an examination setting, making up 4.5% of the final grade. Subsequently, every student took turns leading their team, and their performance was assessed only on one occasion. Two teacher raters were positioned at each ACLS station, and all students were assessed via the same assessment form. Two healthcare professors and eight Doctors of Medicine made up the raters. Additionally, three students from the observing team were asked to rate the team leader of the performing team independently, without discussing their assessments with each other. The students were allocated approximately 10 min to rate their peers during the debriefing session in each station. Their scores accounted for 1.5% of the 4.5%.

### Scoring and rater consistency

The response data were analyzed using *StataCorp 2021’s Stata Statistical Software: Release 17, College Station, TX: StataCorp LLC*. Categorical data were presented as percentages and continuous variables as means with standard deviations (SD) and median with interquartile range (IQR) as appropriate. One-way ANOVA was utilized to compare means across stations to determine differences in difficulty. A two-way mixed-effects model intraclass correlation coefficient (ICC) determined the interrater reliability among the three student raters [35].

### Internal structure

#### Reliability analysis

The assessment tool’s internal reliability was checked using Cronbach’s alpha. To determine the assessment tool’s reliability, a generalizability theory analysis was performed using a three-way ANOVA or a two-faceted, fully crossed random-effect (p × i × r) design. This method employed a comprehensive crossed design involving persons (P), items (I), and raters (R). The analysis aimed to identify the variance in measurements arising from the different facets of the study [36]. Then, the variance components were calculated, and EduG version 6e was used to recheck the calculation [37, 38]. Of note, the analysis covered seven variance components: the main effects of the student’s score (P), items (I), raters (R), and the two-way interactions between score and items (PI), score and raters (PR), and item and raters (IR). Also, it considered the residual error variance (PRI, e), encompassing interactions among all facets and any other unidentified sources of variability [39, 40].

Additionally, a two-facet crossed design decision study was conducted to explore variations in the G-coefficient across different facet conditions and determine the most effective measurement approach. It was important to note that a unique set of raters graded each student, but every rater and student was judged on all items. Thus, a two-faceted nested design (r:(p × i)) was manipulated, where each person (*N* = 95) was valued across all items (*N* = 10). However, the subset of raters (groups of 3 raters) varied for each person-item combination. This setup implied that persons were crossed with items, with raters nested within each person-item combination (Supplementary Fig. 1) [39–41].

The absolute G-coefficient (Phi-coefficient) was adopted to determine the reliability of each facet combination. The Phi-coefficient, which accounted for systematic (main) effects of the facets that might introduce error into the estimate, was included in the error term. This absolute coefficient was chosen because the scores contributing to each student’s grade point average (GPAX) were based on predefined criteria, not relative comparison. The established lower limit for reliability (Phi-coefficient) was 0.70 for formative examination and 0.80 for summative examination, which signified high generalizability of assessment scores [41, 42].

#### Validity analysis

Construct validity was confirmed using confirmatory factor analysis (CFA), conducted using *StataCorp 2021’s Stata Statistical Software: Release 17, College Station, TX: StataCorp LLC*. The maximum likelihood extraction was utilized. Additionally, basic assumptions for sampling adequacy, such as multicollinearity and data normality, were weighed using the Kaiser–Meyer–Olkin (KMO) measure. The model’s fit was determined through six indices: (1) chi-square test, *χ*^2^; (2) chi-square test to degrees of freedom ratio, *χ*^2^/df; (3) comparative fit index (CFI); (4) Tucker-Lewis index (TLI); (5) root mean square error of approximation (RMSEA); and (6) standardized root mean square residual (SRMR). Collectively, these indices suggested a satisfactory model fit. Specifically, a *χ*^2^/df ratio below 3, CFI and TLI values over 0.90, and RMSEA and SRMR values below 0.08 all indicated an acceptable fit between the observed data and the proposed model [43, 44].

### Relations to other variables

Criterion-related validity was done by comparing the peer ratings to the teacher ratings. An independent *t*-test compared mean scores between peer and teacher raters. Pearson’s correlation gauged interrater reliability between average teacher and student ratings. The rater agreement between the two was also calculated [35].

### Consequences

#### Pass rates

The consequential aspect of construct validity pertains to evaluating a test’s short-term and long-term impacts. Scores derived from a valid assessment instrument can potentially enhance learning and teaching [45]. Cut-off scores are determined by the minimum passing threshold, represented by a 60% aggregate. Students who score below 6 on any item will receive feedback during the debriefing session to aid their improvement and ensure they meet the established criteria.

#### Student’s perceptions of peer assessment

Perceptions of peer assessment within the simulation-based context were gathered through a questionnaire administered after the course. This questionnaire comprised ten items rated on a 5-point Likert scale, aimed at determining students’ perceptions of the advantages of peer assessment in terms of enhancing their understanding of learning objectives, readiness for learning, motivation, group participation, communication, leadership, critical thinking, confidence in expressing opinions, congruence with learning objectives, assessment of skills, and facilitation of the exchange of ideas and feedback.

## Results

### Content

Based on the IOC appraised by three experts, all items scored above 0.50, ranging from 0.67 to 1.00. The experts recommended modifications to the descriptions of the affective domain rubrics, primarily focusing on specifying the rubric criteria and simplifying them for improved clarity (Fig. 2). As an illustration, when it came to the communication skill items, the experts recommended the inclusion of explicit details regarding the specific communication skills that students needed to demonstrate, encompassing aspects such as building rapport and effectively conveying information.

### Response process

Among 95 students, nineteen participated in stations 2, 4, and 5, eighteen in station 3, and twenty in station 1. Each station received two teacher ratings (*N* = 190) and three peer ratings (*N* = 285). Average ratings across stations ranged from 9.34 to 9.53 with no significant differences (*p* = 0.479), indicating consistent difficulty levels, as detailed in Table 1.

**Table 1:** Scores of the integration of basic EKG interpretation into the ACLS station across stations

Station	Mean ± SD	Median (IQR)	Levene statistic (*p*-value)	F	*p*-value
Station 1 (*n* = 20)	9.49 ± 0.36	9.61 (9.29–9.73)	0.433 (0.784)	0.880	0.479
Station 2 (*n* = 19)	9.34 ± 0.33	9.31 (9.14–9.66)			
Station 3 (*n* = 18)	9.45 ± 0.33	9.45 (9.12–9.71)			
Station 4 (*n* = 19)	9.53 ± 0.26	9.59 (9.36–9.70)			
Station 5 (*n* = 19)	9.45 ± 0.36	9.49 (9.38–9.68)			

*SD* Standard deviation, *EKG* Electrocardiogram, *ACLS* Advanced cardiac life support

#### Internal consistency reliability and inter-rater reliability among peer raters

Three peer raters assessed ninety-five third-year preclinical medical students at PCM in a single ACLS examination session. The average scores in the first domain (EKG and ACLS algorithm skills) were 9.46 ± 0.81, 9.41 ± 0.65, and 9.38 ± 0.73 for the first to third raters, respectively (Table 2). In the second domain (scenario management and mechanism of action), the average scores were 9.38 ± 0.82, 9.44 ± 0.65, and 9.32 ± 0.75. In the third domain (affective domain), the average scores were 9.47 ± 0.89, 9.54 ± 0.71, and 9.40 ± 0.82 for the first to third raters, respectively. The ICC for the first to third domains were 0.76, 0.61, and 0.71, respectively (*p* = 0.001 for all). Overall, the ICC was 0.78 (*p* = 0.001).

**Table 2:** Interrater reliability of the peer assessment

Domain	Rater 1	Rater 2	Rater 3	ICC	*p*-value
	Mean ± SD	Mean ± SD	Mean ± SD		
EKG and ACLS algorithm skills	9.46 ± 0.81	9.41 ± 0.65	9.38 ± 0.73	0.76	0.001
Scenario management and mechanism of action	9.38 ± 0.82	9.44 ± 0.65	9.32 ± 0.75	0.61	0.001
Affective domain	9.47 ± 0.89	9.54 ± 0.71	9.40 ± 0.82	0.71	0.001
Total	9.44 ± 0.73	9.46 ± 0.54	9.37 ± 0.64	0.78	0.001

*SD* Standard deviation, *EKG* Electrocardiogram, *ACLS* Advanced cardiac life support, *ICC* Intraclass correlation coefficient

### Internal structure

#### Reliability analysis

The Cronbach’s alpha for peer ratings was 0.83. Table 3 elucidates the outcomes of the two-faceted G-study for p × i × r and nested r:(p × i) designs, focusing on the SBL course’s overall score as assessed by peer raters. The data indicates that 20.10% of the total variance is attributed to the universe score of students (P). The variance component, which includes the interaction of students (P) with the number of assessment tool items (I), accounts for 11.24%. In the nested design, the variance components attributed to students and items are 24.41% and 1.20%, respectively. The presence of student-item interaction yields an impact on the overall variance, amounting to 7.03%. In contrast, the residuals encompass a greater proportion of the percentage (67.35%). Hence, these findings strongly imply that the student’s performance is the primary source of variance in the overall score. Conversely, the influence of each assessment item on the variation is relatively negligible.

**Table 3:** Generalizability study for p × i × r and nested r:(p × i) designs using peers as raters among 95 preclinical medical students, ten items, and three raters

Source of variation p × i × r design	% of the total variance	Source of variation r:(p × i) design	% of the total variance
**Student (P)**	20.10	**Student (P)**	24.41
**Item (I)**	1.26	**Item (I)**	1.20
**Rater (R)**	0.03		
**PI**	11.24	**PI**	7.03
**PR**	12.81		
**IR**	0.00	**Rater (R):PI**	67.35
**Residual (PIR, e)**	54.57		
**Total**	100.00		100.00

Table 4 shows the decision study for the p × i × r design, forecasting the reliability for different combinations of assessment items and student raters. Using a ten-item rubric, the Phi-coefficient ranges from 0.51 to 0.73 for one to three raters. In the p × i × r design, eight items with three raters are necessary to achieve acceptable reliability (Phi-coefficient ≥ 0.70). On the other hand, the nested r:(p × i) design indicates that only one rater with a ten-item rubric is needed to reach acceptable reliability (Phi-coefficient = 0.76). When adopting two raters, the Phi-coefficient fluctuates from 0.74 to 0.90 for 5 to 15 items and from 0.80 to 0.92 for three raters. Therefore, to obtain good reliability (Phi-coefficient ≥ 0.80), either two peer raters through eight items (Phi-coefficient = 0.82) or one rater via fifteen items (Phi-coefficient = 0.83) are required.

**Table 4:** Decision study of a p × i × r and r:(p × i) design for assessment of the integration of simulation-based learning into clinically correlated electrocardiogram interpretation and ACLS course

Effect	Estimate variance components in D-study												
	**n**_**r**_**′**	**1**	**1**	**1**	**1**	**2**	**2**	**2**	**2**	**3**	**3**	**3**	**3**
	**n**_**i**_**′**	**5**	**8**	**10**	**15**	**5**	**8**	**10**	**15**	**5**	**8**	**10**	**15**
**p × i × r design**													
E*p*^2^		0.44	0.49	0.51	0.54	0.59	0.64	0.66	0.69	0.66	**0.72**	**0.74**	**0.76**
Φ		0.43	0.49	0.51	0.54	0.58	0.64	0.66	0.69	0.66	**0.71**	**0.73**	**0.76**
**r:(p × i) design**													
Ep^2^		0.62	**0.72**	**0.77**	**0.83**	**0.75**	**0.83**	**0.86**	**0.90**	**0.81**	**0.87**	**0.89**	**0.93**
Φ		0.62	**0.72**	**0.76**	**0.83**	**0.74**	**0.82**	**0.85**	**0.90**	**0.80**	**0.86**	**0.89**	**0.92**

*Ep*^*2*^ G-coefficient, *Φ* Phi-coefficient, *n*_*r*_*′* Number of raters, *n*_*i*_*′* Number of items, *Bold* Reliable assessment of ≥ 0.70

### Validity analysis

**Fig. 3:**
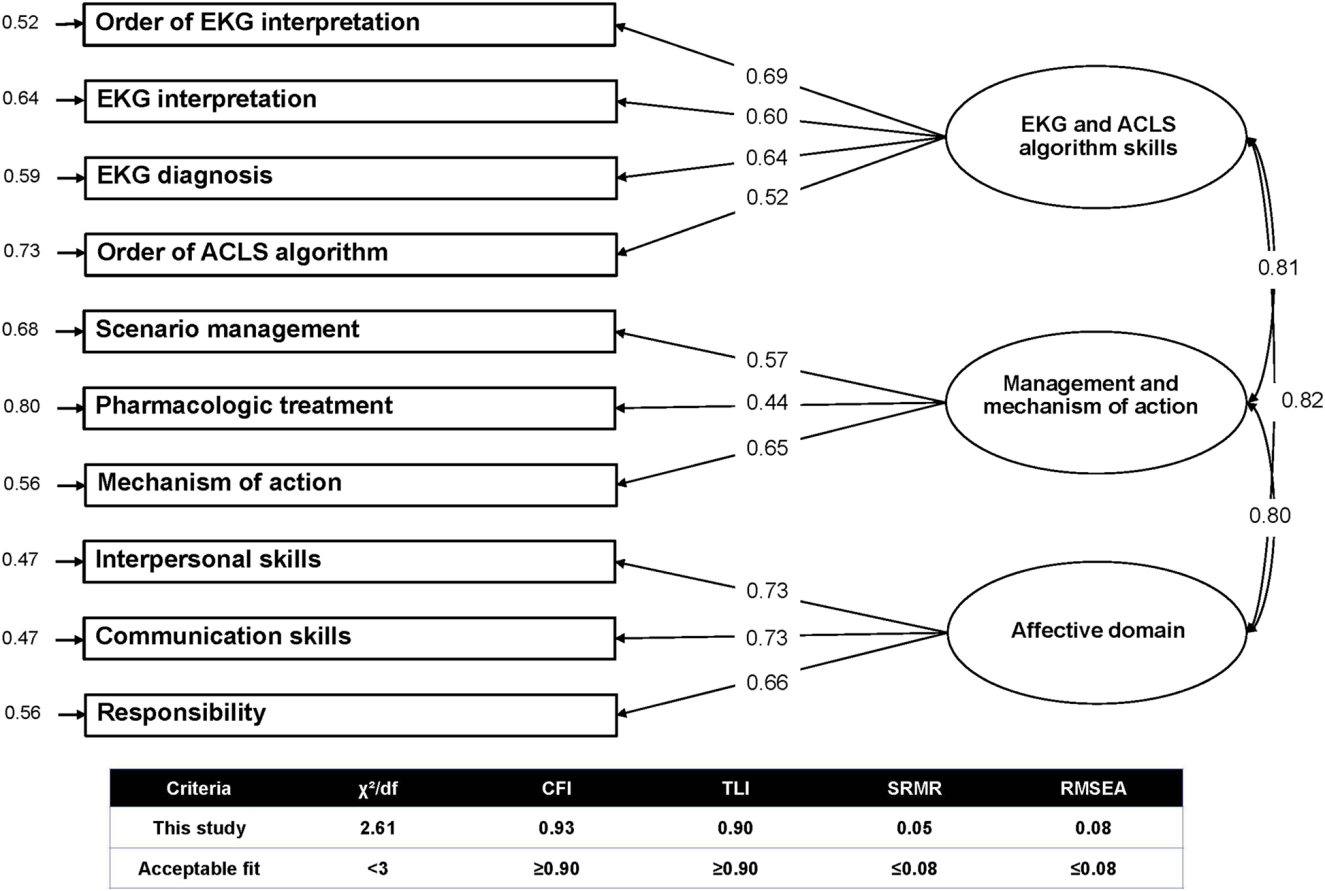
Confirmatory factor analysis of the assessment rubric by peer ratings managed in the integration of simulation-based learning into clinically correlated electrocardiogram interpretation and ACLS course. The model’s fit revealed a normed chi-square (*χ*^2^/df) of 2.61, CFI = 0.93, TLI = 0.90, RMSEA = 0.08, and SRMR = 0.05, highlighting an acceptable fit

The Kaiser–Meyer–Olkin test gave a value of 0.869, and the chi-square for Bartlett’s test of sphericity was significant (*χ*^2^ = 778.52, df = 45, and *p* = 0.001), denoting a sufficiently large sample size for analysis (*N* = 285 ratings). The fit of the model was evaluated, revealing a normed chi-square (*χ*^2^/df) of 2.61, CFI = 0.93, TLI = 0.90, RMSEA = 0.08, and SRMR = 0.05, all of which point to an acceptable fit. In addition, the CFA comprised three domains with ten latent variables extracted from the assessment form. Factor loadings for each item fluctuated from 0.44 to 0.73, as depicted in Fig. 3.

### Relations to other variables

**Fig. 4:**
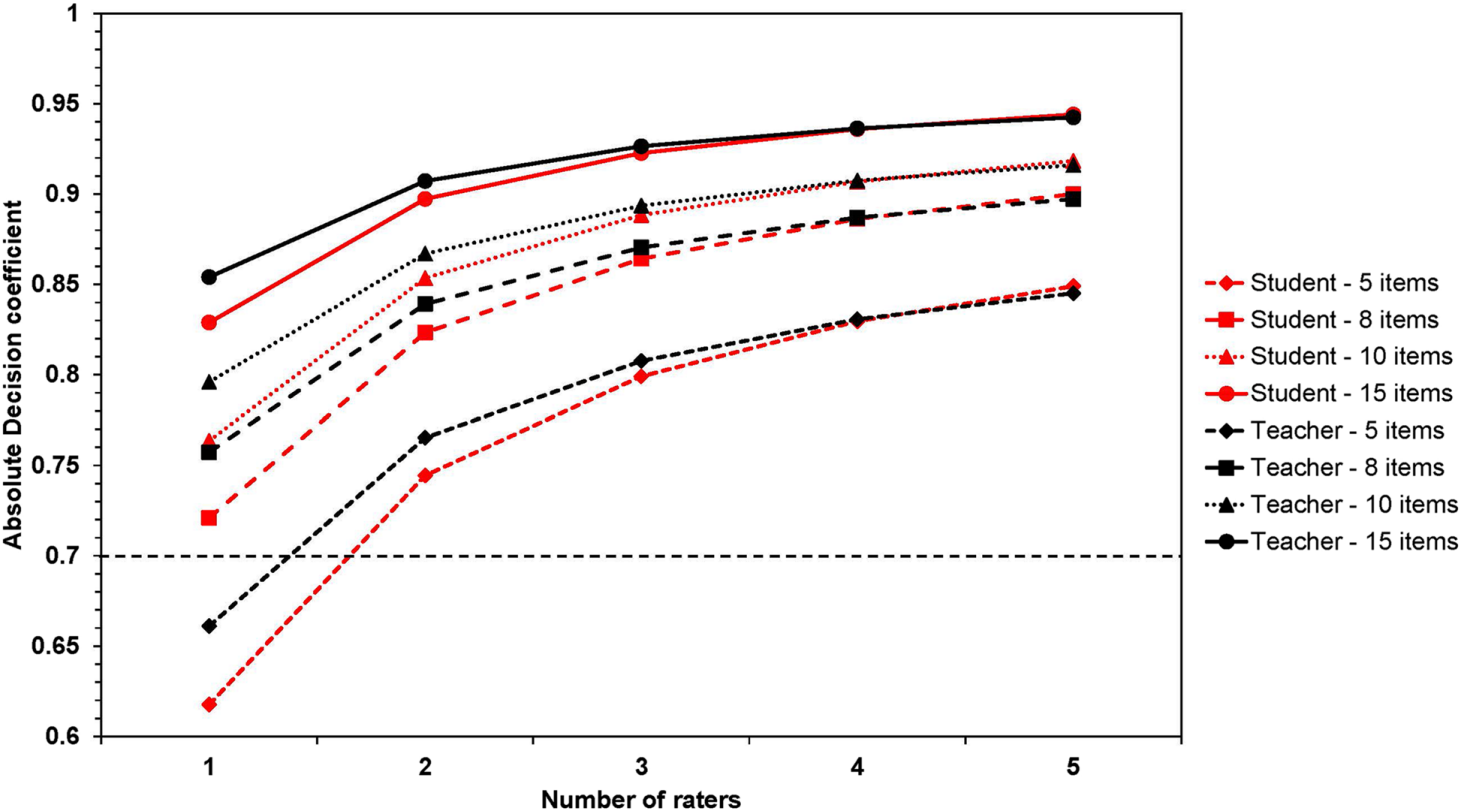
Decision study results for the preclinical medical students (*n* = 95) who were engaged in the integration of simulation-based learning into clinically correlated electrocardiogram interpretation and ACLS course via a ten-item rubric comparing teacher and peer as raters. The coefficients highlight the projected absolute G-coefficient for multiple combinations of items and raters. The dashed line indicates acceptable reliability of phi-coefficient greater than or equal to 0.70

A comparison of average scores between two teacher ratings (gold standard) and three peer ratings was conducted (Table 5). The overall peer and teacher rating scores were 9.42 ± 0.64 and 9.36 ± 0.75, respectively (*t* = 1.04, *p* = 0.150). The peer rating (9.38 ± 0.74) was slightly higher than the teacher rating (9.25 ± 0.87) in the scenario management and mechanism of action domain (*t* = 1.70, *p* = 0.045). Moreover, the interrater reliability between peer and teacher ratings was 0.454 (*p* = 0.001) (Supplementary Fig. 2). The Bland–Altman plot showed good agreement between peer and teacher ratings for scores above 90 out of 100 (Supplementary Fig. 3). Table 6 exhibits the agreement between average teacher and peer ratings. In the first domain, 76 (80.00%) ratings were in exact agreement with a discrepancy of 1 (1.05%). In the second and third domains, there were 62 (65.26%) and 69 (72.63%) exact agreements, respectively, each with 4 (4.21%) discrepant ratings. Additionally, Fig. 4 displays a nested r:(p × i) decision study that contrasts the reliability of teacher and student ratings. The analysis uncovers that although student ratings exhibit marginally lower reliability, an equal number of student and teacher raters are necessary for attaining commendable reliability with the same quantity of items.

**Table 5:** Comparison of the average scores between the teacher and the peers rating on the assessment of the integration of simulation-based learning into clinically correlated electrocardiogram interpretation and ACLS course

Domain	Peers	Teacher	*t*	*p*-value
	**Mean ± SD**	**Mean ± SD**		
**EKG and ACLS algorithm skills**	9.42 ± 0.73	9.33 ± 0.89	1.12	0.132
**Scenario management and mechanism of action**	9.38 ± 0.74	9.25 ± 0.87	1.70	0.045
**Affective domain**	9.47 ± 0.81	9.49 ± 0.78	− 0.22	0.587
**Total**	9.42 ± 0.64	9.36 ± 0.75	1.04	0.150

*SD* Standard deviation, *EKG* Electrocardiogram, *ACLS* Advanced cardiac life support

**Table 6:** Rater agreement of the average scores in each domain between the teacher and the peers rating on the assessment of the integration of simulation-based learning into clinically correlated electro-cardiogram interpretation and ACLS course

Domain	EKG and ACLS algorithm skills				Scenario management and mechanism of action				Affective domain			
**Average teacher rating**	**Average peer rating**				**Average peer rating**				**Average peer rating**			
	< 8	8 to 8.99	9 to 10	Total	< 8	8 to 8.99	9 to 10	Total	< 8	8 to 8.99	9 to 10	Total
< 8	**2**	3	1	6	**1**	1	3	5	**0**	1	2	3
	**2.11%**	3.16%	1.05%	6.32%	**1.05%**	1.05%	3.16%	5.26%	**0.00%**	1.05%	2.11%	3.16%
8 to 8.99	1	**2**	11	14	1	**2**	12	15	1	**1**	6	8
	1.05%	**2.11%**	11.58%	14.74%	1.05%	**2.11%**	12.63%	15.79%	1.05%	**1.05%**	6.32%	8.42%
9 to 10	0	3	**72**	75	1	15	**59**	75	2	14	**68**	84
	0.00%	3.16%	**75.79%**	78.95%	1.05%	15.79%	**62.11%**	78.95%	2.11%	14.74%	**71.58%**	88.42%
Total	3	8	84	95	3	18	74	95	3	16	76	95
	3.16%	8.42%	88.42%	100.00%	3.16%	18.95%	77.89%	100.00%	3.16%	16.84%	80.00%	100.00%

*EKG* Electrocardiogram, *ACLS* Advanced cardiac life support, *Bold* Exact agreement between raters

### Consequences

#### Pass rates

All students achieved scores above 60%, with the lowest average score at 74.5%. However, a teacher assigned a rating of 4 to a student in EKG interpretation, EKG diagnosis, and ACLS algorithm. Similarly, a peer assigned a rating of 4 to another student in EKG interpretation, resulting in feedback during the debriefing session.

#### Student’s perceptions of peer assessment

Most students hold positive perceptions of peer assessment, as depicted in Supplementary Fig. 4. Over 90% strongly agreed or agreed that peer assessment facilitates a better understanding of learning objectives, aids in planning and preparation, boosts motivation, encourages group participation, and fosters the exchange of ideas and feedback. Meanwhile, approximately 80 to 90% concurred that it promotes effective communication, enhances leadership skills, cultivates critical thinking, enables confident opinion expression, and aligns with and accurately assesses learning objectives and skills.

## Discussion

This study assesses the validity and reliability of assessment rubrics via peer ratings among third-year preclinical students in an SBL environment, focusing on integrating EKG interpretation into an ACLS station course. The study describes the rubric’s development and the administration process and gathered validity evidence following Messick’s validity framework.

Validity evidence supporting the peer assessment rubric is summarized and discussed as follows:

### Content

The rubric in the current study is comprehensively aligned with the course learning objectives. Its content was validated through various methods, including the application of the AMEE guiding framework to assist in the rubric development and alignment with the Thai Medical Competency Assessment Criteria. Three experts conducted a review of the content utilizing the IOC method. Experts suggested detailing the specific skills students should exhibit within the affective domains. For instance, communication skills include building rapport and effectively conveying information. This approach could improve raters’ ability to assess affective skills, which are typically challenging accurately. Additionally, alpha, beta, and pilot testing were performed to guarantee the rubric’s robustness.

### Response process

The present study has established acceptable levels of interrater reliability. The ICC among peer raters across all domains was found to be satisfactory. The results demonstrated that the ratings from each peer assessor were consistent, aligning with the minimal variance observed among raters in the generalizability study. The satisfactory inter-rater reliability observed may stem from the extensive preparation of peer raters, which included detailed explanations supplemented by examples. Nonetheless, conducting a formative assessment prior to the full course implementation could further enhance the reliability.

### Internal structure

#### Reliability analysis

The internal consistency reliability was good, revealing that the tool consistently measured the intended construct across items, ensuring reliable feedback and accurately identifying strengths and areas for improvement, while the generalizability theory analysis assists in supporting the reliability of peer assessments.

Previous research has used the generalizability theory to determine the variance in divergent instructional and learning models, identifying potential error sources in measurement conditions across the universe of observations [41]. Also, similar studies focusing on performance-based assessments with scoring rubrics frequently reported significant unexplained residual variance [36, 41, 46]. To the best of our knowledge, this study was the first to use generalizability theory analysis to assess ACLS simulation-based scenario scores among preclinical students with student raters. As such, direct comparisons with similar studies were not possible.

Previous generalizability theory research in preclinical years has emphasized problem-based learning. In contrast, SBL studies have primarily concentrated on residency programs rather than preclinical medical students [26, 47]. Moreover, SBL courses encountered limitations due to the high number of students relative to the available mannequins, raters, and time [27]. Nevertheless, this study’s analysis suggested that only one student rater was needed to obtain a reliable assessment.

While a single rater on a one-time assessment might yield satisfactory reliability, it was recommended that students be engaged in more assessments to advance their peer rating abilities. As noted by Harden, frequent involvement in peer assessment could be crucial for students in today’s medical education landscape [48]. Evidently, regular calibration exercises and feedback could assist raters in consistently applying criteria and raising their confidence in their ratings [49, 50]. Furthermore, increased involvement in assessment processes and student participation in rubric development could heighten assessment quality and future performance [51].

#### Validity analysis

The construct validity in this study is acceptable, denoting that each item properly represents its respective domain. The inherent challenge in crafting each criterion is providing ample informative guidance for its creation and scoring while ensuring it does not overwhelm the reader or performer. Overwhelming them could undermine the construct validity, which is essential to maintain [17, 52]. Assessing performance-based and affective domains like interpersonal skills and student responsibility requires comprehensive yet concise criteria [53]. This need for balance is evident and is suggested in the instructors’ content validation of the current study. Although rubrics show clear performance guidelines and expectations, ensure consistent grading, and provide specific feedback, poorly designed rubrics can lead to misconceptions and reduced learning effectiveness [17].

### Relations to other variables

The interrater reliability between the average ratings of students and teachers was moderate (*r* = 0.45, *p* = 0.001). In a meta-analysis of peer assessment on digital platforms since 1999, Li et al. (2016) discovered an average correlation of 0.63 between instructor and peer ratings in a summative setting [16]. Moreover, the agreement between the comprehensive ratings of students and teachers was poor for students scoring below 90, which might be attributed to the study’s summative context. Consequently, there might be a tendency for friendly biased marking among those with lower performance. Overall, peers tended to assign higher marks than teachers in cognitive domains, like EKG and ACLS algorithm skills, scenario management, and mechanism of action. Discrepancies in scoring were most observed in the scenario management and mechanism of action domain. However, in the affective domain, peer and teacher assessments were consistent [28].

According to prior research comparing teacher and peer assessments in SBL, peer observers typically scored lower on cognitive outcomes than facilitators [27]. This disparity could arise because students were assigned self-directed learning tasks on topics such as ACLS administration, resulting in less practical experience compared to instructors. Consequently, students might not accurately identify subpar work, leading to a tendency towards lenient grading due to friendly bias. This issue could be addressed by showcasing a range of student performances, from high achievers to those who struggled, illustrating the marking criteria for each. This approach would offer students a more comprehensive understanding of the rubrics. Additionally, incorporating practice rounds of simulation-based learning or formative examinations could enhance student expertise before the assessment [54].

### Consequences

As evidenced in this study, the students’ favorable perceptions towards the peer assessment process align with existing literature that suggests peer assessment can effectively engage students and enhance their motivation to learn [15, 50]. This potential for positive outcomes should inspire educators to explore further and implement peer assessment strategies. However, it is important to note that the current study did not capture data on other significant consequences, such as student short- and long-term competency and confidence; these aspects warrant attention in future research.

A practice formative examination is recommended to improve peer assessment’s reliability and validity and boost students’ confidence and knowledge before summative examinations. Frequent peer assessments and structured feedback can amplify the rate at which students receive feedback, thereby improving their performance in future clinical and academic pursuits [55–57]. These assessments not only refine students’ feedback-giving skills but also reinforce their teamwork and communication abilities [58].

### Strengths and future implications

This study offers valuable insights into the validity and reliability of peer assessment rubrics, including the optimal number of peer raters needed for reliable assessments. One student rater suffices for formative exams, whereas summative exams require two. A distinct rater group for each student in the nested r:(p × i) model reveals a similar necessary number of peers and teacher raters for reliable assessments. Furthermore, student assessments generally align with teacher ratings despite a tendency for students to overrate cognitive skills. This study result could be implemented in several settings, including peer rating and feedback in a formative setting, which is a viable alternative for teachers. Incorporating a peer-led formative examination can lessen the number of teachers required compared to traditional formative examinations. Hence, the present study provides awareness and strategies for employing rubrics in the ACLS simulation-based learning context.

### Limitations

Firstly, the study focused on third-year preclinical students from a specific educational setting, limiting the generalizability of its findings due to the context-dependent nature of generalizability theory in medical education SBL settings [41]. Further external validation is needed to assess applicability across distinct educational contexts, academic levels, clinical settings, and cultures. Secondly, the generalizability study model in the present study did not account for variables such as the number of test occasions and the sequence of student examinations, which could influence complete scores. This limitation arose because students could only be assessed once due to constraints related to the availability of raters and time. Lastly, there may be scope for broader data collection regarding the consequences of the construct validity evidence. The present study only gathered perceptions of peer assessment; other outcomes, such as confidence levels or long-term real-life assessment, were not collected. Moreover, the cutoff points might require revision through various methods, such as the modified Angoff or Ebel method, for standard setting prior to future courses.

## Conclusion

This study successfully demonstrates the validity and reliability of rubrics used in a simulation-based learning environment, focusing on integrating EKG interpretation into an ACLS station learning, employing peers as raters. The rubrics from this study could aid in future peer assessments and feedback, enhancing students’ competencies in EKG interpretation within ACLS contexts. A single rater, whether a peer or a teacher, is sufficient for reliable assessment to achieve acceptable reliability. However, multiple rounds of assessment could enhance students’ abilities as assessors, improving their skills and accuracy in assessing their peers.

### Supplementary Information

Supplementary Material 1.

## Data Availability

The datasets used and/or analyzed during the current study are available by reasonable request from the author via Sethapong.ler@pcm.ac.th.

## Abbreviations

SBL: Simulation-based learning
ACLS: Advanced cardiac life support
PCM: Phramongkutklao College of Medicine
