# Supplementary material for: Development of peer assessment rubrics in simulation-based learning for advanced cardiac life support skills among medical students
Source: Adv Simul (Lond). 2024 Jun 24;9:25. doi: 10.1186/s41077-024-00301-7 (PMC11194909; doi:10.1186/s41077-024-00301-7)
Supplement: Supplementary file 1 — Supplementary Material 1. [file 41077_2024_301_MOESM1_ESM.docx]

**Supplementary File**

| Supplementary Figure 1. Sources of variability of nested r:(p×i) design  Supplementary Figure 2. Scatter plot between average student ratings and teacher ratings. | Page 2  Page 3 |
| --- | --- |
| Supplementary Figure 3. The Bland-Altman plot shows the agreement between average student ratings and teacher ratings. | Page 4 |
| Supplementary Figure 4. Student’s perception toward peer assessment | Page 5 |
| Supplementary Table 1. Reporting Guidelines for Health Care Simulation Research Extensions to the CONSORT Checklist | Page 6-10 |

**Supplementary** **figure 1.** Sources of variability of nested r:(p×i) design. Each student (N=95) is figured out by all items (N=10), but the subset of the raters (3 raters) is different for each combination of persons and items. A person is crossed with items. Raters are nested within the combination of persons and items.

**
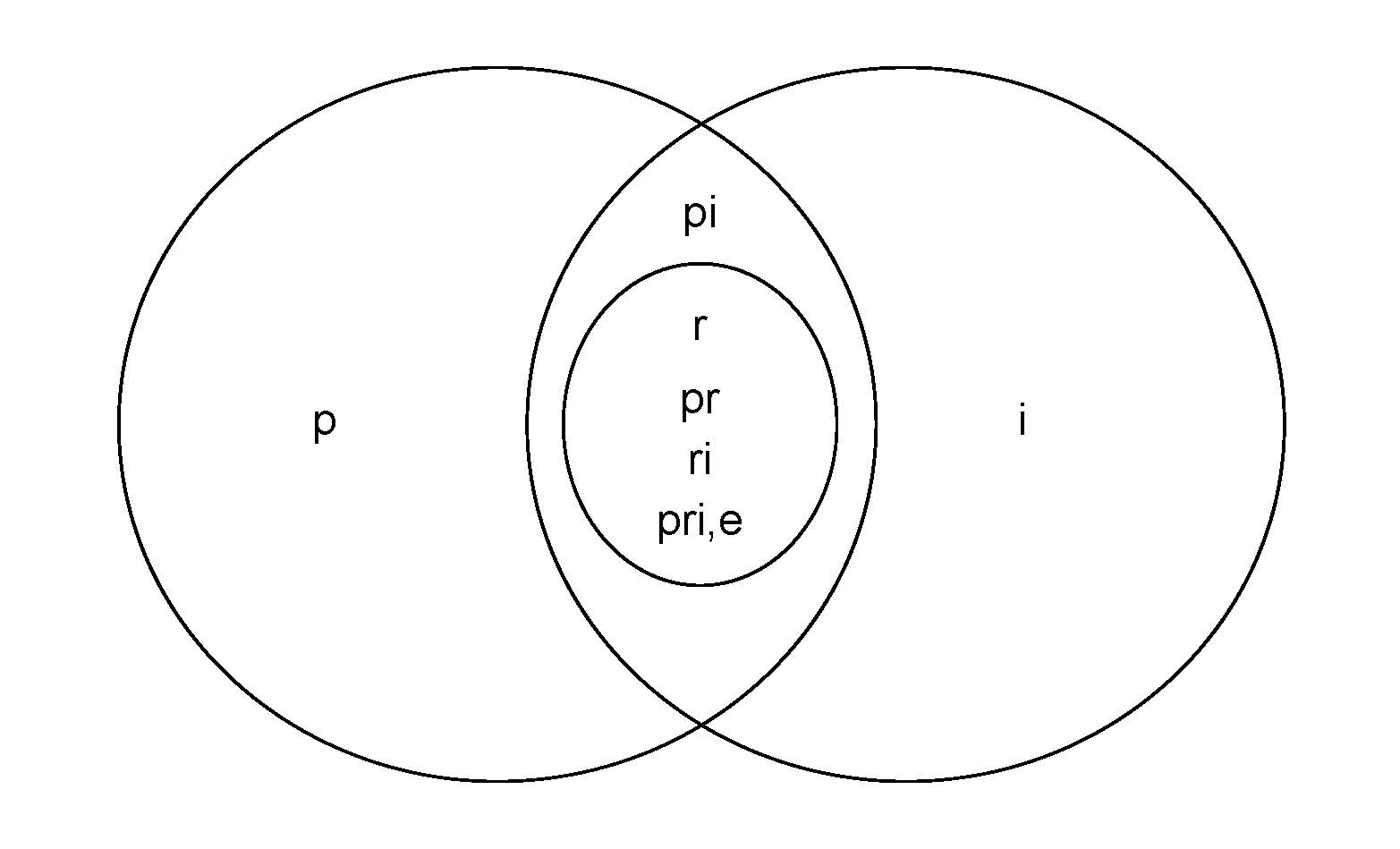
**

**Supplementary figure 2.** Scatter plot between average student ratings and teacher ratings.

**
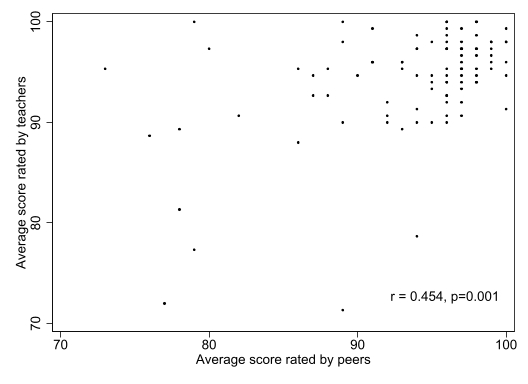
**

**Supplementary figure 3.** The Bland-Altman plot shows the agreement between average student ratings and teacher ratings.


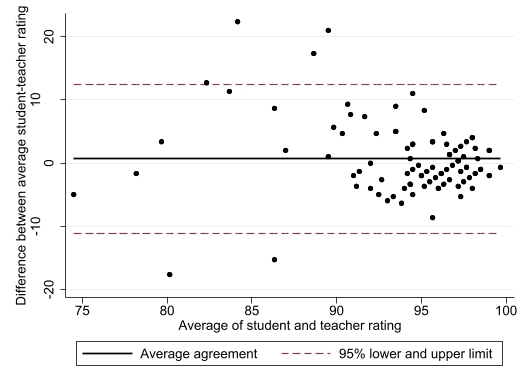


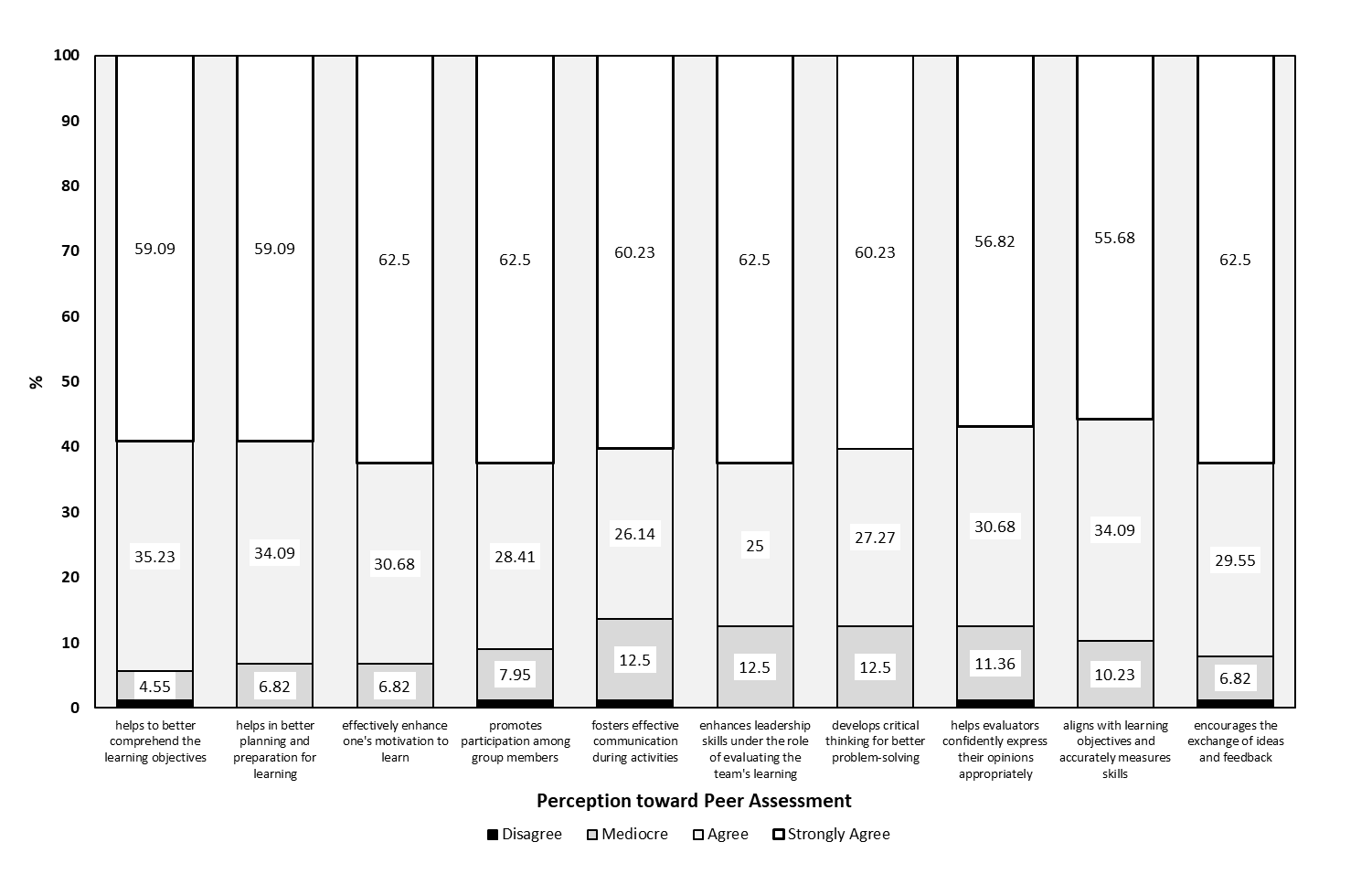
**Supplementary figure 4.** Student’s perception toward peer assessment

| Supplementary table 1. Reporting Guidelines for Health Care Simulation Research Extensions to the CONSORT Checklist | | | | | |
| --- | --- | --- | --- | --- | --- |
| Section/Topic | | Item No | Checklist item | Extension for Simulated-Based research | Reported on page No |
|  | Title and abstract | | | | |
|  | | 1a | Identification as a randomised trial in the title | In abstract or key terms, the MESH or searchable keyword term must have the word ‘‘simulation’’ or ‘‘simulated.’’ | 2 |
|  |  | 1b | Structured summary of trial design, methods, results, and conclusions (for specific guidance see CONSORT for abstracts) |  | 2 |
|  | Introduction | | | | |
| Background and objectives | | 2a | Scientific background and explanation of rationale | Clarify whether simulation is subject of research or investigational method for research. | 6 |
|  |  | 2b | Specific objectives or hypotheses |  | 6 |
|  | Methods | | | | |
| Trial design | | 3a | Description of trial design (such as parallel, factorial) including allocation ratio |  | 9-10 |
|  |  | 3b | Important changes to methods after trial commencement (such as eligibility criteria), with reasons |  | N/A |
| Participants | | 4a | Eligibility criteria for participants |  | 7 |
|  |  | 4b | Settings and locations where the data were collected |  | 7 |
| Interventions | | 5 | The interventions for each group with sufficient details to allow replication, including how and when they were actually administered | Describe the theoretical and/or conceptual rationale for the design of each intervention. Clearly describe all simulation-specific exposures, potential confounders, and effect modifiers. | 7 |
| Outcomes | | 6a | Completely defined pre-specified primary and secondary outcome measures, including how and when they were assessed | In describing the details of methods of assessment, include (when applicable) the setting, instrument, simulator type, timing in relation to the intervention, along with any methods used to enhance the quality of measurements. Provide evidence to support the validity and reliability of assessment tools in this context (if available). | 7-10 |
|  |  | 6b | Any changes to trial outcomes after the trial commenced, with reasons |  | N/A |
| Sample size | | 7a | How sample size was determined |  | 7 |
|  |  | 7b | When applicable, explanation of any interim analyses and stopping guidelines |  | N/A |
| Randomisation: | |  |  |  |  |
| Sequence generation | | 8a | Method used to generate the random allocation sequence |  | 9 |
|  |  | 8b | Type of randomisation; details of any restriction (such as blocking and block size) |  | N/A |
| Allocation concealment mechanism | | 9 | Mechanism used to implement the random allocation sequence (such as sequentially numbered containers), describing any steps taken to conceal the sequence until interventions were assigned |  | N/A |
| Implementation | | 10 | Who generated the random allocation sequence, who enrolled participants, and who assigned participants to interventions |  | N/A |
| Blinding | | 11a | If done, who was blinded after assignment to interventions (for example, participants, care providers, those assessing outcomes) and how | Describe strategies to decrease risk of bias, when blinding is not possible. | N/A |
|  |  | 11b | If relevant, description of the similarity of interventions |  | N/A |
| Statistical methods | | 12a | Statistical methods used to compare groups for primary and secondary outcomes | Clearly indicate the unit of analysis (eg, individual, team, system), identify repeated measures on subjects, and describe how these issues were addressed. | 9-10 |
|  |  | 12b | Methods for additional analyses, such as subgroup analyses and adjusted analyses |  | N/A |
|  | Results | | | | |
| Participant flow (a diagram is strongly recommended) | | 13a | For each group, the numbers of participants who were randomly assigned, received intended treatment, and were analysed for the primary outcome |  | N/A |
|  |  | 13b | For each group, losses and exclusions after randomisation, together with reasons |  | N/A |
| Recruitment | | 14a | Dates defining the periods of recruitment and follow-up |  | N/A |
|  |  | 14b | Why the trial ended or was stopped |  | N/A |
| Baseline data | | 15 | A table showing baseline demographic and clinical characteristics for each group | In describing characteristics of study participants, include their previous experience with simulation and other relevant features as related to the intervention(s). | 9-10 |
| Numbers analysed | | 16 | For each group, number of participants (denominator) included in each analysis and whether the analysis was by original assigned groups |  | 14-15 |
| Outcomes and estimation | | 17a | For each primary and secondary outcome, results for each group, and the estimated effect size and its precision (such as 95% confidence interval) | For assessments involving >1 rater, interrater reliability should be reported. | 14-15 |
|  |  | 17b | For binary outcomes, presentation of both absolute and relative effect sizes is recommended |  | 16 |
| Ancillary analyses | | 18 | Results of any other analyses performed, including subgroup analyses and adjusted analyses, distinguishing pre-specified from exploratory |  | 14-16 |
| Harms | | 19 | All important harms or unintended effects in each group (for specific guidance see CONSORT for harms) |  | N/A |
|  | Discussion | | | | |
| Limitations | | 20 | Trial limitations, addressing sources of potential bias, imprecision, and, if relevant, multiplicity of analyses | Specifically discuss the limitations of SBR | 23 |
| Generalisability | | 21 | Generalisability (external validity, applicability) of the trial findings | Describe generalizability of simulation-based outcomes to patient-based outcomes (if applicable). | 22 |
| Interpretation | | 22 | Interpretation consistent with results, balancing benefits and harms, and considering other relevant evidence |  | 17-21 |
| Other information | | | |  |  |
| Registration | | 23 | Registration number and name of trial registry |  | N/A |
| Protocol | | 24 | Where the full trial protocol can be accessed, if available |  | N/A |
| Funding | | 25 | Sources of funding and other support (such as supply of drugs), role of funders | List simulator brand and if conflict of interest for intellectual property exists. | 25 |

The table is adapted from CONSORT2010 Statement: updated guidelines for reporting parallel group randomized trials^1^ and the Reporting Guidelines for Health Care Simulation Research

Extensions to the CONSORT and STROBE Statements^2^.

1. Cheng A, Kessler D, Mackinnon R, Chang TP, Nadkarni VM, Hunt EA, et al. Reporting guidelines for health care simulation research: extensions to the CONSORT and STROBE statements. Advances in Simulation. 2016;1:25.

2. Schulz KF, Altman DG, Moher D. CONSORT 2010 Statement: updated guidelines for reporting parallel group randomised trials. BMC Med. 2010;8:18.
